# An exploratory cross-sectional study of subclinical vascular damage in patients with polymyalgia rheumatica

**DOI:** 10.1038/s41598-020-68215-8

**Published:** 2020-07-09

**Authors:** Rossana Scrivo, Valeria Silvestri, Francesco Ciciarello, Paola Sessa, Iolanda Rutigliano, Cristina Sestili, Giuseppe La Torre, Cristiana Barbati, Alessio Altobelli, Cristiano Alessandri, Fulvia Ceccarelli, Manuela Di Franco, Roberta Priori, Valeria Riccieri, Antonio Sili Scavalli, Francesca Romana Spinelli, Luciano Agati, Francesco Fedele, Bruno Gossetti, Fabrizio Conti, Guido Valesini

**Affiliations:** 1 Department of Clinical Internal, Anesthesiological and Cardiovascular Sciences, Rheumatology Unit, Sapienza University of Rome, viale del Policlinico 155, 00161 Rome, Italy; 2 Department of General Surgery, Surgical Specialities “Paride Stefanini”, Sapienza University of Rome, Rome, Italy; 3 Department of Public Health and Infectious Diseases, Sapienza University of Rome, Rome, Italy; 4 Policlinico Umberto I di Roma, Rome, Italy

**Keywords:** Cardiology, Vasculitis syndromes, Risk factors

## Abstract

The aim of the study was to investigate the presence of subclinical vascular damage in polymyalgia rheumatica (PMR). We enrolled PMR patients having major cardiovascular risk factors (MCVRF) and, as controls, patients with MCVRF. All underwent: color Doppler ultrasound to evaluate the common carotid intima-media thickness (IMT), the anterior–posterior abdominal aortic diameter (APAD), and the prevalence of carotid artery stenosis; the cardio-ankle vascular index (CAVI) to measure arterial stiffness together with the ankle-brachial index (ABI) to investigate the presence of lower-extremity peripheral arterial disease. Finally, we measured the serum levels of adipocytokines implicated in vascular dysfunction. As a result, 48 PMR and 56 MCVRF patients were included. An increase of IMT (1.07/0.8–1.2 vs 0.8/0.8–1.05; p = 0.0001), CAVI (8.7/7.8–9.3 vs 7.6/6.9–7.8; p < 0.0001) and APAD values (21.15/18.1–25.6 vs 18/16–22; p = 0.0013) was found in PMR patients with respect to controls. No differences were reported in the prevalence of carotid artery stenosis or ABI values between the two groups. A significant correlation between IMT and CAVI in PMR and MCVRF subjects (r^2^ = 0.845 and r^2^ = 0.556, respectively; p < 0.01) was found. Leptin levels (pg/mL; median/25th–75th percentile) were higher in PMR than in MCVRF subjects (145.1/67–398.6 vs 59.5/39.3–194.3; p = 0.04). Serum levels of adiponectin (ng/mL) were higher in PMR patients (15.9/10.65–24.1 vs 6.1/2.8–22.7; p = 0.01), while no difference in serum levels of resistin (ng/mL) was found between PMR and MCVRF subjects (0.37/0.16–0.66 vs 0.26/0.14–1.24). Our study shows an increased subclinical vascular damage in PMR patients compared to those with MCVRF, paving the way for further studies aimed at planning primary cardiovascular prevention in this population.

## Introduction

The association of chronic inflammatory rheumatic disorders such as rheumatoid arthritis (RA), spondyloarthritis, gout, systemic lupus erythematosus (SLE), and scleroderma with an increased risk of vascular disease, especially cardiovascular and cerebrovascular disease, is a consolidated matter^1–8^. Polymyalgia rheumatica (PMR) is a chronic, inflammatory disorder occurring in individuals at the age of 50 years or older, characterized by aching and stiffness in the proximal regions of the extremities as well as elevated markers of inflammation^9^. Incidence increases with age, peaks between 70 and 80 years, and is greater in women that are affected 2–3 times more frequently than men^9^. PMR has been associated, both in men and women, with a 2.6 increased risk for vascular events, including cardiovascular, cerebrovascular, and peripheral vascular events^10^. While in patients with RA and SLE the greatest cardiovascular risk occurs in established disease, in PMR the excess risk was seen both early in the disease and throughout the follow-up period^11^. The relevance of this issue is further supported by the observation that cardiovascular comorbidities in patients with PMR were mainly responsible for an increase in direct medical costs compared to age- and sex-matched controls from the same community in a Minnesota County^12^. However, a systematic review found inconsistent results, with some studies reporting a positive association and others no association between PMR and vascular disease^13^.

The aim of our study was to investigate the presence of subclinical vascular damage in patients with PMR through a validated and non-invasive approach, including the common carotid intima-media thickness (IMT) assessment^14^ and the cardio-ankle vascular index (CAVI), a method for estimating arterial stiffness and predicting cardiovascular risk developed by measuring pulse wave velocity (PWV) and blood pressure^15^.

## Methods

### Data source and study cohort

Between May 2017 and May 2018, consecutive Caucasian patients with PMR according to the EULAR classification criteria^16^ also having major cardiovascular risk factors (MCVRF), including hypertension, diabetes, hypercholesterolemia, cigarette smoking, and obesity, were recruited from the Rheumatology Unit at Sapienza University of Rome, Rome, Italy. As controls, consecutive Caucasian patients with MCVRF were enrolled at the Angiology Unit at Sapienza University of Rome, Rome, Italy, as long as a PMR diagnosis had been excluded.

At recruitment, data on demographics, disease duration, treatments (with particular focus on glucocorticoids for PMR patients) were obtained by direct questioning and collected in a computerized form. Hypertension was defined as values ≥ 140 mmHg systolic blood pressure (SBP) and/or ≥ 90 mmHg diastolic blood pressure (DBP) according to the criteria published in 2013 by the European Society of Hypertension (ESH) and European Society of Cardiology (ESC)^17^. Diabetes was defined on the basis of fasting plasma glucose levels ≥ 126 mg/dL on 2 separate occasions^18^ or the use of anti-diabetic drugs. In accordance with the 2013 American College of Cardiology/American Heart Association Guidelines (ACC/AHA), hypercholesterolemia was defined as total cholesterol ≥ 220 mg/dL or the use of lipid-lowering drugs^19^. Finally, obesity was defined as a body mass index (BMI) of 30 kg/m^2^ or above^20^. All patients had a color Doppler ultrasound assessment and a CAVI measurement. We also analyzed the serum levels of adipocytokines known to be implicated in vascular dysfunction^21^.

This study was conducted in accordance with the Declaration of Helsinki, and informed consent was obtained from all the patients. The study design was approved by the Ethical Committee of Sapienza University of Rome, Rome, Italy (reference number 4625, protocol 729/17).

### Color Doppler ultrasound assessment

The measurement of common carotid artery IMT is a well-established cardiovascular risk marker, being both a measure of early atherosclerosis and of smooth muscle hypertrophy/hyperplasia^22^. Mannheim Carotid IMT Consensus (2004–2006) established guidelines for IMT assessment and distinction between IMT and atherosclerotic plaque by ultrasound based on localization, natural history, risk factors and predictive value for vascular events^14^.

In all patients we performed a two-dimensional echo color Doppler ultrasound of the common carotid arteries, adopting a high definition vascular echograph (Esaote MyLab 50 X-Vision) in order to detect the IMT and the prevalence of carotid stenosis. Carotid artery color Doppler ultrasound was performed by a 7.5 MHz linear transducer, using an overhead technique, with the examiner seated behind the patient’s head. The patient was positioned supine, with its head slightly extended backwards and turned opposite to the side of interrogation. The degree of carotid artery stenosis, where present, was measured according to the European Carotid Surgery Trial (ECST) criteria^23^. Furthermore, we carried out ultrasound imaging of anterior–posterior abdominal aortic diameter (APAD) to screen for abdominal aortic aneurysm occurrence. Abdominal aorta was assessed by a 3.5 MHz convex transducer in longitudinal and transverse planes from the level of the diaphragm to the bifurcation. Aneurysm was defined as infrarenal arterial enlargement with a maximum diameter ≥ 30 mm associated to loss of arterial wall parallelism^24^.

### Cardio-ankle vascular index

Since arterial stiffness has been associated with atherosclerosis, we also studied patients by CAVI, a blood pressure-independent index that measures the stiffness of the aorta, femoral artery and tibial artery. CAVI is calculated using the formula below enriched with measurements from an electrocardiogram, phonocardiogram, brachial artery waveform, and ankle artery waveform^15^:

$$ CAVI = {\alpha} = {\left( {{2{\text{\th}}}/\Delta {{P}}} \right) x {{  ln }}\left( {{{SBP}}/{{DBP}}} \right){{ PWV^2}}}  \, + \beta $$

where ∆P is SBP–DBP, þ is blood density, PWV is pulse wave velocity (another measure of arterial stiffness), and *a* and *β* are constants. Scale conversion constants are determined so as to match CAVI with PWV using Hasegawa’s method. All measurements and calculations are made together and automatically in VaSera (Fukuda Denshi Co. Ltd. Tokyo, Japan). This equation was derived from Bramwell-Hill’s formula and the stiffness parameter β. CAVI uses blood pressure cuffs with sensors on all four limbs to generate plethysmographs. The cuffs are placed on bilateral upper and lower extremities while the patient is in supine position with the limbs at the same level as the heart, in a comfortable position in a warm room. Measurement of ankle-brachial index (ABI), a tool to assess the presence of lower-extremity peripheral arterial disease^17^, was performed during CAVI measurement. Report from the instrument used for CAVI measurement included ABI index value as an output data. ABI was calculated directly by the instrument by using superior and inferior limb systolic pressure measurement. The mean bilateral (right/left side) CAVI and ABI values were used in our analysis.

### Enzyme-linked immunosorbent assay (ELISA)

The serum levels of leptin, adiponectin, and resistin were measured in PMR patients and controls. Venous blood samples were taken after 12 h of nocturnal fasting, then centrifuged and the serum stored at − 35 °C until analysis was performed by using a commercial ELISA according to the manufacturer’s instructions (Invitrogen Thermo Fisher Scientific, Camarillo, CA, USA).

### Statistical analysis

Descriptive statistics [mean, standard deviation (SD); median 25th–75th percentile] were used to present quantitative variables, whereas percentages and frequencies were generated for qualitative variables. Differences between patients with PMR and MCVRF controls were tested using Student’s t test or Mann–Whitney test when appropriate and χ^2^ test for quantitative and categorical variables, respectively. Bivariate analysis was conducted using the Pearson rho coefficient and Spearman correlation coefficient for quantitative variables. The statistical analysis was carried out using SPSS, release 23.0 (SPSS, Inc., Chicago, IL, USA). The statistical significance was set at p < 0.05. A sample size of at least 18 patients per group was estimated for 80% power by assuming a 5% significance level and considering a 2.6 relative risk for vascular events associated with PMR^10^ and a 50% prevalence of cardiovascular disease in the general population.

## Results

### Cohort characteristics

Forty-eight patients with PMR and 56 with MCVRF were included in the study. The main demographic and clinical data are shown in Table 1. No significant differences between the 2 groups with respect to gender, age, and prevalence of MCVRF were found, except for SBP which was significantly higher in patients with PMR (p = 0.03). When appropriate, patients were in pharmacological treatment (anti-hypertensive drugs, anti-diabetic drugs, lipid-lowering drugs) for their own cardiovascular risk factors.

**Table 1:** Main demographic and clinical data of PMR patients and controls.

	PMR (n = 48)	MCVRF (n = 56)	p value
Sex (F/M)	28/20	30/26	0.626
Mean age, years (SD)	73.96 ± 6.89	71.63 ± 6.89	0.088
Mean disease duration, months (SD)	38.7 ± 32	40.3 ± 28	0.542
Cigarette smoking, n/%	8/16.7	16/28.6	0.151
Mean glucocorticoid daily dose, mg (SD)	7.05 ± 5	–	
Mean cumulative glucocorticoid dose, mg (SD)	5946 ± 1537	–	
Hypertension, n/%	25/52.1	31/55.4	0.738
Mean SBP, mmHg (SD)	135 ± 8	130 ± 8	**0.03**
Mean DBP, mmHg (SD)	81 ± 6	77 ± 5	0.437
Diabetes, n/%	9/18.8	9/16.1	0.719
Hypercholesterolemia, n/%	25/52.1	23/41.1	0.261
Mean BMI (SD)	26.1 ± 3.8	24.8 ± 3.1	0.214
Carotid artery stenosis, n/%	13/27.1	7/12.5	0.051
Mean ABI (SD)	1.10 ± 0.11	1.13 ± 1.10	0.228

The p value in bold is the only statistically significant difference

*PMR* polymyalgia rheumatica, *MCVRF* major cardiovascular risk factors, *yrs* years, *SD* standard deviation, *SBP* systolic blood pressure, *DBP* diastolic blood pressure, *BMI* body mass index, *ABI* ankle-brachial index.

### Color Doppler ultrasound assessment and CAVI

**Figure 1:**
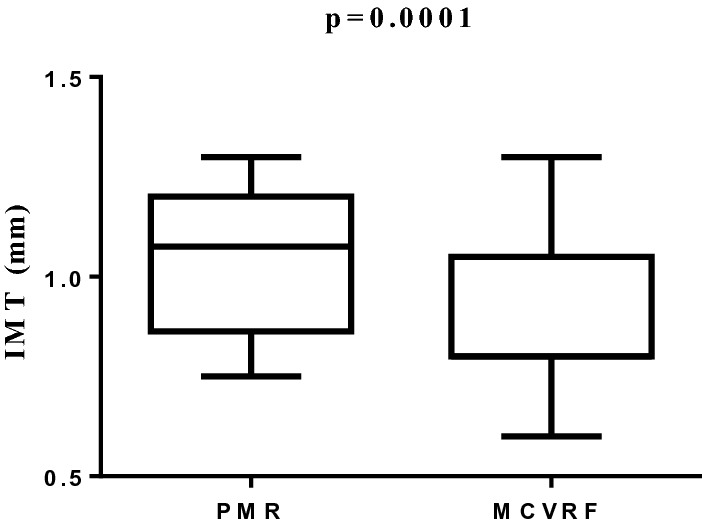
IMT values (right/left side mean values) in patients with PMR (n = 48) and MCVRF controls (n = 56). Data are shown as Tukey boxplots; lines represent the median level with 25th–75th percentile. *IMT* intima-media thickness, *PMR* polymyalgia rheumatica, *MCVRF* major cardiovascular risk factors.

**Figure 2:**
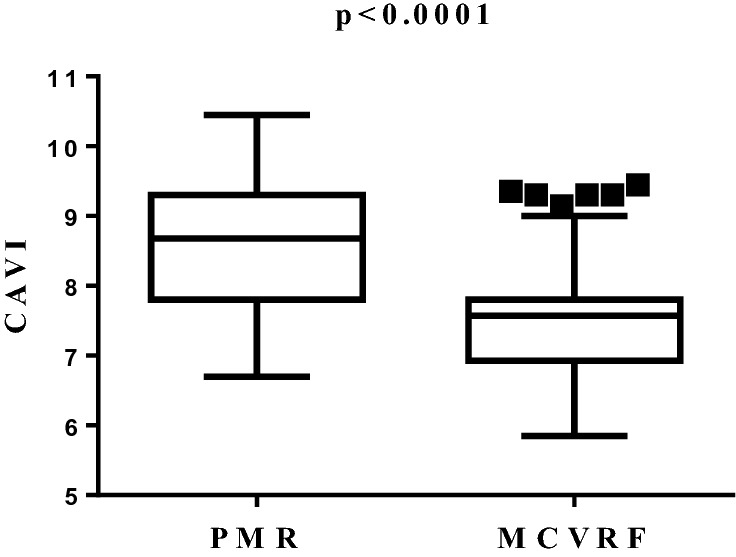
CAVI values (right/left side mean values) in patients with PMR (n = 48) and MCVRF controls (n = 56). Data are shown as Tukey boxplots; lines represent the median level with 25th–75th percentile; data not included between the whiskers are plotted as outliers with dots. *CAVI* cardio-ankle vascular index, *PMR* polymyalgia rheumatica, *MCVRF* major cardiovascular risk factors.

A significant increase of IMT values (median/25th–75th percentile) was found in PMR compared to MCVRF controls (1.07/0.8–1.2 vs 0.8/0.8–1.05; p = 0.0001) (Fig. 1). Likewise, mean CAVI values (median/25th–75th percentile) were higher in patients with PMR than MCVRF controls (8.7/7.8–9.3 vs 7.6/6.9–7.8; p < 0.0001) (Fig. 2).

**Figure 3:**
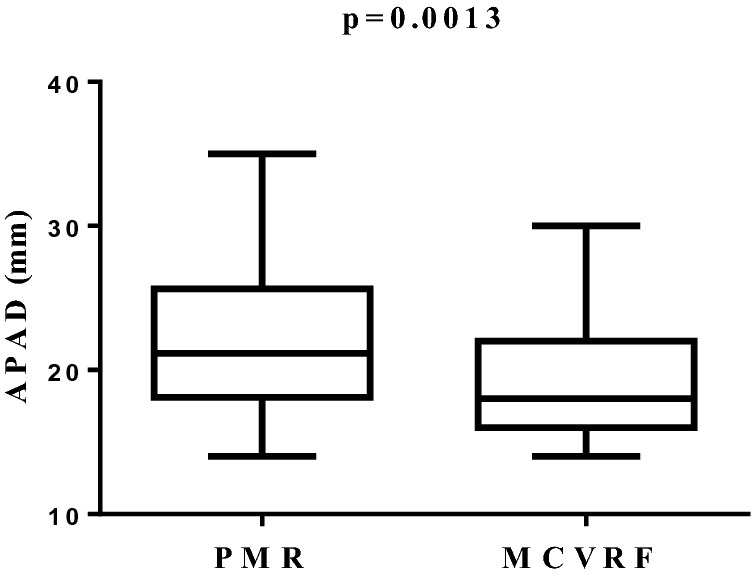
APAD values in patients with PMR (n = 48) and MCVRF controls (n = 56). Data are shown as Tukey boxplots; lines represent the median level with 25th–75th percentile. *APAD* anterior–posterior abdominal aortic diameter, *PMR* polymyalgia rheumatica, *MCVRF* major cardiovascular risk factors.

**Figure 4:**
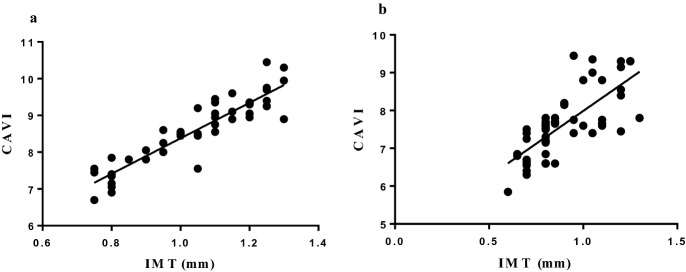
Correlation between IMT and CAVI values (right/left side mean values). (**a**) Patients with polymyalgia rheumatica (n = 48) (${r}^{2}$= 0.845; p < 0.01). (**b**) Major cardiovascular risk factors controls (n = 56) (${r}^{2}$= 0.556; p < 0.01). *CAVI* cardio-ankle vascular index, *IMT* intima-media thickness.

When we evaluated APAD for occurrence of abdominal aortic aneurysm, we found an increased value (median/25th–75th percentile) in patients with PMR compared to MCVRF controls (21.15/18.1–25.6 vs 18/16–22; p = 0.0013), in the absence of aortic aneurysm (Fig. 3). No differences were reported with regards to the prevalence of carotid artery stenosis or ABI values between the two groups. In our study, no patient had an ABI value < 0.9, which would have been an exclusion criterion being potentially responsible for unreliable CAVI values^15^. No significant correlation between disease duration or mean glucocorticoid daily dosage and IMT or CAVI values was found in PMR patients. Finally, the results of the bivariate regression analysis showed a positive correlation between IMT and CAVI values in both PMR patients and MCVRF subjects (${r}^{2}$=0.845 and ${r}^{2}$=0.556, respectively; p < 0.01) (Fig. 4).

### Enzyme-linked immunosorbent assay (ELISA)

**Figure 5:**
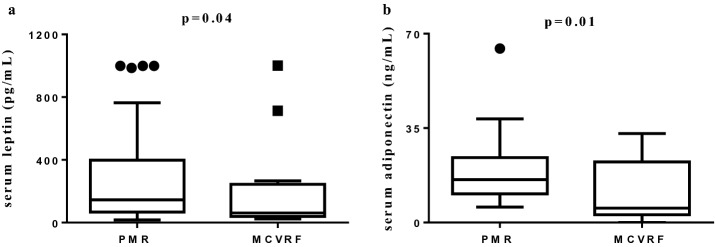
Comparison of adipokines serum levels in patients with PMR (n = 40) and MCVRF controls (n = 18). (**a**) Serum leptin levels. (**b**) Serum adiponectin levels. Data are shown as Tukey boxplots; lines represent the median level with 25th–75th percentile; data not included between the whiskers are plotted as outliers with dots. *PMR* polymyalgia rheumatica, *MCVRF* major cardiovascular risk factors.

Serum levels of leptin, adiponectin, and resistin were measured in 40 patients with PMR and in 17 MCVRF controls. Leptin levels (pg/mL; median/25th–75th percentile) were higher in patients with PMR than in MCVRF subjects (145.1/67–398.6 vs 59.5/39.3–194.3; p = 0.04). Likewise, serum levels of adiponectin (ng/mL) were higher in patients with PMR (15.9/10.65–24.1 vs 6.1/2.8–22.7; p = 0.01) (Fig. 5), while no difference in serum levels of resistin (ng/mL) was found between PMR and MCVRF subjects (0.37/0.16–0.66 vs 0.26/0.14–1.24) (not shown). No correlation was found analyzing serum adipocytokine levels with each other or with the clinical and laboratory parameters of PMR patients and MCVRF subjects.

## Discussion

The high prevalence of traditional cardiovascular risk factors together with the burden of the systemic inflammatory response sustains the increased cardiovascular risk in chronic inflammatory rheumatic diseases^1–8^.

Although a systematic review found inconsistent results with respect to the increased vascular damage in PMR^13^, some studies do support this item^10,25^. Analyzing the electronic database of primary care physicians in England and Wales, a 2.6 relative risk for cardiovascular disease in PMR patients with respect to the general population was reported over a median follow-up period of 7.8 years. In particular, the relative risk was found to be 2.7 for cardioischemic disease, 2.3 for cerebrovascular disease, and 2.8 for peripheral artery disease^10^. Furthermore, a meta-analysis of observational high-quality studies that included 34,569 patients with PMR showed a 72% excess risk of coronary artery disease among patients with PMR compared with subjects without PMR, even if the statistical heterogeneity of the reviewed studies was high^26^. Conversely, in a recent study, patients with PMR or GCA did not have a high risk for cardiovascular disease, but we must remark that the setting was Japan population, having the lowest incidence of PMR in the world and milder symptoms than in the Western countries^27^.

The association of PMR with vascular disease may be explained by the elevated levels of C-reactive protein, which is also one of the independent biomarkers associated with cardiovascular disease^28^. Furthermore, vascular inflammation was demonstrated in most patients with PMR by ^18^F-FDG PET/CT imaging, showing an increased ^18^F-FDG uptake mainly in the aorta and its major branches^24,29^. This finding implies, even in PMR patients without clinical signs of vasculitis such as stenosis or aneurysm, possible subclinical inflammatory lesions on arteries. Interestingly, we found significantly increased values of APAD in the absence of abdominal aortic aneurysm in our patients with PMR, who did not have clinically overt giant cell arteritis, compared to MCVRF controls.

This assessment was part of our study, aimed at assessing the subclinical vascular involvement in PMR patients, in order to obtain data that could eventually suggest the need for planning primary cardiovascular prevention. We performed color Doppler ultrasound to evaluate, in addition to APAD, carotid IMT and carotid artery stenosis; we also assessed CAVI to measure arterial stiffness parameters and ABI. These are considered appropriate tools for detecting asymptomatic organ damage in the context of overall cardiovascular risk due to their non-invasiveness, availability, suitability for outpatients, repeatability and low cost^17^. IMT significantly predicts plaque occurrence^14^ and serves as a proxy of generalized atherosclerosis when a value of > 0.9 mm is found^17^. In PMR patients we observed a significant increase of carotid IMT with respect to controls and, notably, the mean IMT was superior to the 0.9 mm cut-off. Since IMT is a critical marker of increased risk of vascular events, this finding is consistent with the results of previous studies^10,25^ and supports further attention on the risk of cardiovascular disease in PMR. Another parameter for evaluating cardiovascular risk is the arterial stiffness, traditionally assessed in terms of carotid-femoral PWV. Its correlations with carotid IMT, carotid stenosis, aortic and peripheral atherosclerosis have been proved^30^. We decided to investigate this parameter by using an alternative tool, i.e. CAVI, a non-invasive and a blood pressure-independent technique which can be used to estimate the risk of atherosclerosis^31^. Not only our PMR patients had greater mean CAVI score than controls, but also showed a positive correlation with carotid IMT, confirming the potential of CAVI as a useful and reliable cardiovascular risk marker. A similar correlation was found in MCVRF patients. Consistent with these results, CAVI score was positively correlated with the carotid IMT in patients with hypertension^32^, type 2 diabetes mellitus^33^, coronary artery disease^34^, in those with moderate cardiovascular risk^31^, and in the general population^35^.

Glucocorticoids are the recommended first-line treatment in PMR, but their role in promoting an increased cardiovascular risk is controversial. Potential side effects of glucocorticoids, including hypertension, hyperlipidemia, and diabetes mellitus may indeed participate in this adverse outcome^36^. On the other hand, glucocorticoids may decrease the vascular risk in patients with PMR by suppressing inflammation^37^. In fact, a decrease of aortic PWV after prednisone treatment was found in patients with PMR^38^. Accordingly, in our patients neither we found any significant correlation between the mean daily dosage of glucocorticoids (evaluated at the time of the study) and carotid IMT or CAVI values nor between the mean cumulative dosage of glucocorticoids and carotid IMT or CAVI values.

Finally, we measured serum levels of adipocytokines, which are considered biomarkers of cardiovascular risk since their levels are increased in patients with metabolic syndrome, insulin resistance, and obesity^39^. Leptin and resistin showed pro-atherogenic effects under experimental conditions, whereas adiponectin seems to have both anti- and pro-inflammatory activity, probably explained by its different isoform^40^. In RA patients, adiponectin showed critical pro-inflammatory properties, being elevated in serum and synovial fluid, strongly expressed at the synovium level, and directly correlating with disease activity and radiologic progression^41^. Similarly, in our PMR patients we observed significantly higher serum levels of leptin and adiponectin compared to MCVRF controls, which parallels the increased cardiovascular risk demonstrated by ultrasound and CAVI, although no correlation was found with clinical and laboratory features of the patients. Serum levels of leptin and adiponectin had been previously investigated in two studies including a small number of PMR patients, where a significant increase was observed after treatment with glucocorticoids^42,43^. Conversely, glucocorticoids are able to reduce serum levels of resistin, as shown in a small number of patients with systemic autoimmune diseases^44^. Indeed, unlike leptin and adiponectin, resistin is mainly produced by bone marrow-derived mononuclear cells with some contribution from adipocytes^45^. It is conceivable that we did not observe any difference in serum levels of resistin between PMR and MCVRF probably due to the effect of glucocorticoid therapy in patients with PMR.

The main limitation of our study is represented by the cross-sectional design, which does not help to establish a cause-effect ratio between inflammation and vascular lesions. In such a design, the timing of the snapshot does not assure to be representative and a longitudinal follow-up should be prompted to support the data.

In conclusion, we evaluated for the first time the presence of subclinical vascular damage in patients with PMR. Our results show an increase in subclinical cardiovascular lesions with respect to patients with MCVRF, paving the way for further studies aimed at planning primary cardiovascular prevention in PMR patients. If our data will be confirmed in longitudinal studies, the cardiovascular risk profile should be considered as part of the overall management of PMR patients by using validated algorithms which may suggest lifestyle changes and additional treatments.

## Data Availability

The datasets generated and/or analysed during the current study are available from the corresponding author on reasonable request.
